# Unimpaired social cognition in adult patients with ADHD: brain volumetric and behavioral results

**DOI:** 10.1093/scan/nsab060

**Published:** 2021-05-07

**Authors:** Aylin Mehren, Christiane Margarete Thiel, Swantje Bruns, Alexandra Philipsen, Jale Özyurt

**Affiliations:** Department of Psychiatry and Psychotherapy, University Hospital Bonn, Bonn 53127, Germany; Biological Psychology Lab, Department of Psychology, School of Medicine and Health Sciences, Carl von Ossietzky Universität Oldenburg, Oldenburg 26129, Germany; Biological Psychology Lab, Department of Psychology, School of Medicine and Health Sciences, Carl von Ossietzky Universität Oldenburg, Oldenburg 26129, Germany; Research Center Neurosensory Science, Carl von Ossietzky Universität Oldenburg, Oldenburg 26129, Germany; Cluster of Excellence ‘Hearing4all’, Carl von Ossietzky Universität Oldenburg, Oldenburg 26129, Germany; Biological Psychology Lab, Department of Psychology, School of Medicine and Health Sciences, Carl von Ossietzky Universität Oldenburg, Oldenburg 26129, Germany; Department of Psychiatry and Psychotherapy, University Hospital Bonn, Bonn 53127, Germany; Biological Psychology Lab, Department of Psychology, School of Medicine and Health Sciences, Carl von Ossietzky Universität Oldenburg, Oldenburg 26129, Germany; Research Center Neurosensory Science, Carl von Ossietzky Universität Oldenburg, Oldenburg 26129, Germany

**Keywords:** theory of mind, voxel-based morphometry, affective, cognitive, structural MRI, empathy

## Abstract

The present study aimed to investigate whether adult patients with attention deficit hyperactivity disorder (ADHD) show deficits in social cognition and to identify the structural neural correlates of social cognitive skills in ADHD. Twenty-six adult patients with ADHD and 26 matched healthy control participants performed the Movie for the Assessment of Social Cognition and underwent a structural magnetic resonance imaging scan. We compared theory of mind (ToM) performance between ADHD patients and healthy controls. Using voxel-based morphometry, we further compared gray matter volumes in regions that are critical for social cognition between the two groups and examined whether ToM performance was correlated with brain morphometry measures. We did not observe any between-group differences in ToM abilities or regional gray matter volumes. Across both groups, performance on affective aspects of ToM correlated positively with gray matter volumes in the medial part of the superior frontal gyri, which is typically involved in social cognition. This study is the first to relate brain structure to social cognitive abilities in adult patients with ADHD. Although our sample was small and heterogeneous, with half of the patients showing mild-to-moderate psychiatric comorbidities, our results may encourage longitudinal studies that relate social cognitive development in childhood and adolescence to brain maturation of ADHD patients.

## Introduction

Patients with attention deficit hyperactivity disorder (ADHD) are frequently faced with a range of interpersonal difficulties and poor social functioning (Harpin *et al.*, 2016). Besides the core symptoms of ADHD, i.e. inattention, hyperactivity, impulsivity and impaired executive functions, deficits in social cognition might contribute to the development of social interaction problems. Social cognition is a prerequisite for successful social behavior and includes the recognition of emotions, e.g. from facial expressions, body posture or prosody, as well as empathy and more complex operations such as theory of mind (ToM; Frith, 2008; Frith and Singer, 2008). ToM refers to the ability to infer others’ mental states, such as intentions, beliefs and desires (Premack and Woodruff, 1978), and involves cognitive perspective taking (cognitive ToM) as well as affective components (affective ToM) (Shamay-Tsoory and Aharon-Peretz, 2007; Frith and Singer, 2008). Note that definitions of empathy and ToM often overlap. Some authors denote cognitive aspects of empathy as ToM and call affective components of ToM empathy. Here, we base our definition on Premack and Woodruff (1978), where empathy and ToM both refer to imputing a purpose to another individual, but ToM specifically includes the ability to draw inferences about another’s mental states, including cognitive and affective components.

Both cognitive ToM and affective ToM have been repeatedly demonstrated to be impaired in children with ADHD (for reviews, see Uekermann *et al.*, 2010; Bora and Pantelis, 2016). However, even though also adults with ADHD often experience social difficulties (Nijmeijer *et al.*, 2008), studies on social cognitive abilities in adult patients are scarcer and provide heterogeneous results. While some authors have reported similar deficits in children and adults with ADHD (e.g. Rapport *et al.*, 2002; Ibanez *et al.*, 2014; Retz-Junginger *et al.*, 2016), a meta-analysis including a broad range of social cognition tests has come to the conclusion that in adult patients no significant impairments in ToM and only subtle deficits in facial or vocal emotion recognition can be found (Bora and Pantelis, 2016). The authors suggested that social cognition might improve with age, which could be interpreted in the context of a developmental delay in cortical maturation in ADHD patients (e.g. Shaw *et al.*, 2007). A recent study reported deficits in affective but not cognitive perspective taking in a sample of adult patients with ADHD (Abdel-Hamid *et al.*, 2019), suggesting that those two aspects of social cognition might be subject to different developmental processes. The authors argued that only cognitive components of perspective taking might improve in youth with ADHD, while more emotional components might not be subject to compensation.

Brain networks underlying social cognition include fronto-striatal and fronto-limbic regions, such as the medial prefrontal cortex, amygdala, orbitofrontal cortex, anterior cingulate cortex, ventral striatum and insula, as well as temporo-parietal areas including the superior temporal sulcus, temporo-parietal junction, temporal poles and precuneus/posterior cingulate cortex (Hornak *et al.*, 2003; Dalgleish, 2004; Calder *et al.*, 2004; Amodio and Frith, 2006; Kennedy and Adolphs, 2012; Wang *et al.*, 2018; Hiser and Koenigs, 2018). Structural and functional abnormalities have been reported in fronto-striatal, fronto-limbic and temporo-parietal brain regions in children and adult patients with ADHD (Dickstein *et al.*, 2006; Schneider *et al.*, 2006; Makris *et al.*, 2007; Paloyelis *et al.*, 2007; Cubillo and Rubia, 2010; Arnsten and Rubia, 2012; Wolfers *et al.*, 2017; Gao *et al.*, 2019). However, there is also evidence suggesting that brain abnormalities in ADHD patients diminish with increasing age (Shaw *et al.*, 2007; Nakao *et al.*, 2011; Frodl and Skokauskas, 2012; Hoogman *et al.*, 2017, 2019). It is also not yet clear how neural alterations in ADHD may contribute to potential impairments in social cognitive skills.

A recent study indicated an association between cortical thickness and subcortical brain volumes with social skills in children and adolescents with ADHD (aged from 6 to 18 years) (Baribeau *et al.*, 2019). Thinner cortices in regions involved in mentalizing (e.g. superior temporal gyrus, temporal poles, temporo-parietal junction, dorsomedial prefrontal cortex, posterior cingulate cortex and insula) and decreased amygdala and striatal volumes were associated with more deficits in social interaction as rated by patients’ caregivers using the Social Communication Questionnaire. In addition, worse ToM performance (as assessed by the Reading the Mind in the Eyes test) was associated with smaller amygdala and hippocampal volumes. In addition, an exploratory analysis revealed that the relation between brain structural measures and social deficits was less pronounced at older ages.

The aim of the present study was to investigate to which extent social cognitive abilities are impaired in adult patients with ADHD and to examine their structural neuroimaging correlates. Patients and matched healthy controls performed the Movie for the Assessment of Social Cognition (MASC; Dziobek *et al.*, 2006), which is an ecologically valid video-based tool that presents real-life interactions. It has been shown to be sensitive to even subtle ToM deficits and to provide higher discriminative power compared to standard ToM tasks (e.g. Reading the Mind in the Eyes test), while minimizing the possibility of social desirability bias (Dziobek *et al.*, 2006; Korner *et al.*, 2009). The MASC allows to identify deviations in ToM in two directions, overly simplistic (reduced) and overly complex (exceeding) mental state inferences; cognitive and affective ToM abilities can also be considered separately (Dziobek *et al.*, 2006; Montag *et al.*, 2010, 2011). In addition, participants underwent a high-resolution structural magnetic resonance imaging (MRI) scan. Using voxel-based morphometry (VBM), we compared gray matter volumes in regions that are critical for social cognition between ADHD patients and healthy controls and examined whether social cognition performance is correlated with brain morphometric measures.

## Materials and methods

### Participants

Data for the current study were recorded within the context of a larger project focusing on the effects of physical exercise on executive functions of ADHD patients and healthy controls (see Mehren *et al.*, 2019a,b,c). For the present study, all patients who had participated in the larger project were included. In total, data of 26 adult patients with ADHD [median age: 31.0, interquartile range (IQR): 14.25, 5 females] and their respective 26 age- and gender-matched healthy controls (median age: 28.5, IQR: 13.25, 5 females) were analyzed. The sample size was determined based on an a priori power analysis using G*Power 3.1 (Faul *et al.*, 2007) for the primary outcome parameters of the larger project. Assuming small-to-moderate effect sizes, which have been reported in previous studies for exercise effects in ADHD, a sample of 26 patients and 26 healthy controls would yield a power of at least 80% at an alpha level of 0.05.

Recruitment was performed as described by Mehren *et al.* (2019b,c): Adults with ADHD were recruited through the specialist outpatient clinic for adult ADHD of the Department of Psychiatry and Psychotherapy at the University of Oldenburg, Germany. Before study inclusion, they had received a recent diagnosis of ADHD in adulthood according to international guidelines (National Institute for Health and Care Excellence, 2008; AWMF, 2018), based on the diagnostic criteria of the Diagnostic and Statistical Manual of Mental Disorders (4th ed.; DSM-IV; American Psychiatric Association, 1994). Patients were diagnosed by a specialist psychiatrist trained for diagnosis of ADHD, who was supervised by a second expert for ADHD in adulthood. Diagnosis was based on a detailed clinical and psychosocial interview that integrates somatic differential diagnosis, the patients’ psychiatric and developmental history and observer reports. Six patients had received their first diagnosis of ADHD in childhood and were diagnosed again in adulthood before study inclusion. The remaining 20 patients received their first diagnosis in adulthood. However, consistent with DSM-IV diagnostic criteria for adult ADHD, all patients had already shown ADHD symptoms during childhood. This was confirmed using validated self- and informant-reports [school reports, parents’ reporting, the Wender Utah Rating Scale (WURS-k)]. To further assess ADHD symptoms, patients completed the following questionnaires: the German versions of the ADHD Self Rating Scale (ADHS-SB) (Rösler *et al.*, 2008), WURS-k (Retz-Junginger *et al.*, 2002) and the Conners’ Adult ADHD Rating Scale–Self-Report: Long Form (CAARS-S:L) (Conners *et al.*, 1999). Note that although diagnosis was conducted according to DSM-IV criteria, based on ADHS-SB, all patients also fulfilled the research criteria for ADHD of the International Statistical Classification of Diseases and Related Health Problems (10th ed.; ICD-10; World Health Organization, 2004). Healthy control participants were recruited via announcements in the internet.

Patients and healthy controls had to fulfill the following inclusion citeria: (i) absence of neurological disorders, (ii) absence of autism spectrum disorders, (iii) no intake of psychotropic drugs (for patients: no psychotropic drugs different from medication for ADHD), (iv) suitability for MRI and (v) absence of severe psychiatric disorders (e.g. severe affective or anxiety disorders, schizophrenia, substance abuse and posttraumatic stress disorder), as assessed using the German versions of the Structured Clinical Interview for DSM-IV (SCID-I) (Wittchen *et al.*, 1997), the SCID-II screening questionnaire for personality disorders (First *et al.*, 1997) and the Beck Depression Inventory (BDI-II) (Beck *et al.*, 1996). Patients with mild-to-moderate depression and/or anxiety disorders as well as one patient with a mild form of borderline personality disorder, which are very frequent comorbid disorders in adult ADHD (Kessler *et al.*, 2006; Cumyn *et al.*, 2009; Matthies and Philipsen, 2014), were included (see Table 1 for description of comorbidities). Please note that during the diagnostic process, we carefully made sure that ADHD is the primary diagnosis. Patients who were taking stimulant medication were required to withdraw from their medication at least 48 hours before participating. Table 1 depicts clinical and demographic characteristics of participants.

**Table 1.** Demographic and clinical characteristics among patients with ADHD and healthy controls

Variable	ADHD median (IQR)	Controls median (IQR)	*U*-statistic^a^	*P*-value
Age (years)	31.0 (14.25)	28.5 (13.25)	324.0	0.80
BMI (kg/m^2^)	25.4 (5.7)	23.7 (4.2)	244.0	0.09
Years of education	13.0 (2.6)	15.75 (3.0)	238.5	0.06
MWT-B	28.0 (5.25)	29.0 (6.0)	240.5	0.11
ADHS-SB	32.0 (15.25)	5.0 (5.25)	1.0	<0.001*
WURS-k	44.5 (16.5)	10.0 (13.25)	16.5	<0.001*
CAARS-S:L (T-scores)				
Inattention/memory problems	72.5 (20.75)	–	–	–
Hyperactivity/restlessness	69.5 (18.25)	–	–	–
Impulsivity/emotional lability	67.5 (21.0)	–	–	–
Problems with self-concept	69.5 (25.75)	–	–	–
DSM-IV inattentive symptoms	74.0 (28.25)	–	–	–
DSM-IV hyperactive/impulsive	70.0 (16.75)	–	–	–
DSM-IV total symptoms	73.5 (26.75)	–	–	–
ADHD index	77.0 (15.5)	–	–	–
BDI	10.0 (7.75)	2.0 (3.25)	96.5	<0.001*
Go/no-go sensitivity	3.4 (1.0)	3.4 (0.8)	302.0	0.51
Flanker interference (ms)	77 (37)	82 (20)	287.0	0.47
Stimulant medication^b^	*n* = 4	–	–	–
Comorbid psychiatric disorders	*n* = 14			
Mild or moderate depression	*n* = 13	–	–	–
Anxiety disorder	*n* = 3	–	–	–
Borderline personality disorder	*n* = 1	–	–	–
Employment status				
Employed	*n* = 17	*n* = 6	–	–
Student	*n* = 7	*n* = 19	–	–
Unemployed	*n* = 2	*n* = 1	–	–

IQR = interquartile range; BMI = body mass index.

* *P* < 0.05.

a Mann–Whitney *U* test; ADHD: *N* = 26 (5 females); Controls: *N* = 26 (5 females); includes completed qualifications: primary school 4 years, secondary school 5–9 years depending on diploma, vocational training (includes school education in Germany) 2–4 years, university degree typically 3 years for bachelor’s degree and additional 2 years for master’s degree.

b Current medication, discontinued 48 h prior to each visit.

The study was approved by the ethics committee of the University of Oldenburg. Written informed consent was obtained from all participants prior to study participation.

### Experimental procedure

#### Experimental tests

##### Movie for the Assessment of Social Cognition.

The MASC is an ecologically valid video-based psychometric test for social-cognitive skills that has been shown to be sensitive to even subtle ToM deficits, while minimizing the possibility of social desirability bias (Dziobek *et al.*, 2006; Korner *et al.*, 2009). The movie consists of short sequences showing four young people meeting for a dinner, with a particular focus on the social interaction between the actors. In between the sequences, participants are asked in total 45 questions related to the actors’ mental states, requiring them to understand the actors’ thoughts, feelings or intentions. Each question is presented along with four possible answers in a multiple-choice format, of which one has to be chosen. Besides one correct answer, three distractors are presented, reflecting three types of errors in mental state reasoning: overly simplistic mental state reasoning (less ToM) and complete lack of mental state inferences (no ToM), which are both forms of undermentalizing (reduced ToM), and overly complex mental state inferences (exceeding ToM or overmentalizing). The MASC includes questions related to cognitive ToM (e.g. understanding of thoughts and intentions; 23 questions) and affective ToM (e.g. inferring affective states; 19 questions). Classification of items according to their mental state modality was conducted as described by Montag *et al.* (2011). To assess non-social inferencing, six control questions are in addition included, requiring complex reasoning abilities to be answered correctly. Participants performed the MASC in a self-paced manner. Completion took approximately 30 min.

##### Executive functions and verbal intelligence.

As part of the larger project, all participants completed two executive functioning tasks (inside the MR scanner, directly following performance in the MASC): a Go/No-go task to measure response inhibition and a flanker task to assess interference control (for a detailed description of the tasks, see Mehren *et al.*, 2019b,c). To better characterize our study sample and because previous studies have identified a relation between executive functions and social cognition performance (e.g. Mary *et al.*, 2016), we decided to include data on performance in those two tasks in the current study. Table 1 depicts the sensitivity index *d*' (standardized difference between proportion of hits and false alarms) as indicator of inhibitory performance in the Go/No-go task, and the interference score (difference in reaction times between incongruent and congruent trials) as a measure of interference control in the flanker task. To assess verbal intelligence, participants completed the Multiple Choice Vocabulary Test (MWT-B) (Lehrl, 2005).

#### MRI acquisition.

Due to acquisition of a new research scanner and end of contract with the old scanner facilities, we had to switch to the new scanner after measurement of six patients and their matched healthy controls. Imaging data were obtained in two different 3-Tesla MRI Scanners (Siemens MAGNETOM Verio, 12-channel head array, and Siemens MAGNETOM Prisma, 64-channel head array; Siemens AG, Erlangen, Germany). To control for possible image differences, each patient and his/her respective matched healthy control were tested in the same scanner (Verio: *n* = 6 per group, Prisma: *n* = 20 per group) and scanner was included as a covariate in the statistical models for MRI analyses. High-resolution T1-weighted structural images were recorded using magnetization-prepared rapid gradient-echo (MPRAGE) sequence (1 mm^3^ isotropic voxels, 176 slices, Field-of-View (FoV) = 250 × 250 mm, Repetition Time (TR) = 1900 ms, Echo Time (TE) = 2.52 ms, Flip Angle (FA) = 90°).

### Data analysis

#### Behavioral data analysis.

Behavioral data were analyzed using SPSS Statistics 22 (IBM, Armonk, NY, USA) and JASP 0.13.1. As most outcome variables were not normally distributed (Kolmogorov–Smirnov test), we conducted non-parametric tests and report their median values and IQRs. To ensure comparability to other studies, mean values and standard deviations are in addition reported for all behavioral outcomes, and demographic and clinical data in Supplementary Material. To test for group differences, Mann–Whitney *U* tests were used. To provide further evidence for the presence (alternative hypothesis) or absence (null hypothesis) of group differences, additional Bayesian Mann–Whitney *U* tests with default prior option (Cauchy distribution) were calculated.

Our primary aim was to investigate whether adults with ADHD show deficits in social cognition. Therefore, we defined the sum of MASC answers indicating less or no ToM (reduced ToM) as one of our primary outcome measures. Based on the study by Abdel-Hamid *et al.* (2019) indicating deficits in affective but not cognitive aspects of social cognition in adult ADHD patients, we additionally considered scores for affective ToM and cognitive ToM (number of correct responses for each mental state modality) separately. For those three outcome measures, we applied Bonferroni correction to account for multiple comparisons (significance level of *P* ≤ 0.017). For a complete picture, the number of answers reflecting exceeding ToM, the total number of correct responses and the number of correctly answered control questions are also displayed (see Table 2).

**Table 2.** Performance in the Movie for the Assessment of Social Cognition (MASC) among patients with ADHD and healthy controls

Variable	ADHD median Interquartile Range (IQR)	Controls median (IQR)	*U*-statistic^a^	*P*-value	*r*	BF_10_^b^
Reduced Theory of Mind (ToM)	5.5 (6.0)	6.0 (4.0)	321.5	0.76	−0.04	0.28
Affective ToM	15.0 (3.25)	15.0 (5.0)	326.0	0.83	−0.03	0.29
Cognitive ToM	17.5 (3.25)	18.0 (3.5)	335.5	0.96	−0.01	0.29
Exceeding ToM	5.0 (2.25)	4.0 (3.5)	325.5	0.82	−0.03	0.29
Correct ToM	35.0 (7.25)	35.5 (7.25)	332.0	0.91	−0.02	0.31
Correct control questions	5.0 (1.0)	5.0 (2.0)	293.0	0.39	−0.12	0.39

a Mann–Whitney *U* test.

b Bayesian Mann–Whitney *U* test; ADHD: *N* = 26 (5 females); Controls: *N* = 26 (5 females).

To further explore the relation between ToM and ADHD symptoms, we calculated Spearman rank order correlations between ToM performance and ADHD questionnaire scores (ADHS-SB and WURS-k) across both groups. In addition, we tested for correlations between ToM performance and executive functions. For those exploratory analyses, no adjustment for multiple testing was applied (significance level of *P* ≤ 0.05).

#### VBM analysis.

To examine structural correlates of social cognition performance, we performed VBM analyses using the high-resolution T1-weighted structural MR images (Ashburner and Friston, 2000). Those analyses were conducted based on the tutorial by Ashburner (https://www.fil.ion.ucl.ac.uk/~john/misc/VBMclass15.pdf) using SPM12 (Wellcome Trust Centre for Neuroimaging, London, UK) and MATLAB R2016a. First, raw data were visually inspected to check for artifacts (e.g. stemming from movement), which led to the inclusion of all images for further analyses. As part of the preprocessing, each T1 image was segmented into different tissue types (gray matter, white matter and cerebrospinal fluid). To enhance inter-subject alignment, a Dartel (diffeomorphic anatomical registration through exponentiated lie algebra) template, which was registered to Montreal Neurological Institute (MNI) space, was generated. This template was then used to align the individual spatially normalized scans into MNI space. To preserve regional differences in the absolute amount of gray matter (i.e. gray matter volume), volume change correction (modulation) was conducted by modulating image intensity of each voxel with the Jacobian determinants derived from spatial normalization. Finally, to increase signal-to-noise ratio, images were spatially smoothed using an isotropic Gaussian kernel at 8 mm full width half maximum. After each preprocessing step, images were reviewed visually. In addition, quality control procedures integrated in the Computational Anatomy Toolbox—CAT12 (Gaser and Dahnke, 2016) were applied, indicating good-to-satisfactory data quality for all images before and after preprocessing (weighted overall image quality rating, IQR: for patients between 73% and 86%, median 83%; for healthy controls between 73% and 86%, median 83%; no group differences: *U* = 310.0, *P* = 0.61).

For the purpose of group analyses, total intracranial volumes were computed by summing up gray matter, white matter and cerebrospinal fluid for each volume and were then accounted for by using proportional scaling. This approach is dividing the preprocessed data by the total intracranial volumes. Instead of removing variance explained by total intracranial volume, all data are proportionally scaled by their total intracranial volume values. To investigate volumetric differences between ADHD patients and healthy controls, we conducted two-sample *t*-tests on the preprocessed gray matter images. To test for relations between social cognition and gray matter volumes, we performed correlational analyses between scores reflecting ToM performance (reduced ToM, affective ToM and cognitive ToM) and gray matter volumes across groups, as well as in patients and controls separately. In all group analyses, an absolute threshold masking of 0.2 was used in order to remove edge effects between gray and white matter. As data were acquired in two different MRI scanners, scanner was included as a covariate in all statistical models.

All analyses were conducted hypothesis-driven within predefined regions of interest (ROIs), which have been related to social cognition in previous studies (Hornak *et al.*, 2003; Dalgleish, 2004; Calder *et al.*, 2004; Amodio and Frith, 2006; Kennedy and Adolphs, 2012; Wang *et al.*, 2018; Hiser and Koenigs, 2018). Using the WFU PickAtlas v3.0 (ANSIR Laboratory), a mask was created, which included the following brain regions of the AAL ROILibrary (Tzourio-Mazoyer *et al.*, 2002; Maldjian *et al.*, 2003): medial prefrontal cortex, amygdala, orbitofrontal cortex, anterior cingulate cortex, insula, superior temporal sulcus, temporal poles and precuneus/posterior cingulate cortex, as well as the ventral striatum from the IBASPM toolbox (Alemán-Gómez *et al.*, 2006). An ROI including the temporo-parietal junction was added using a sphere based on peak coordinates reported in previous functional and structural neuroimaging studies (Saxe and Wexler, 2005; Adams *et al.*, 2010; Wurm *et al.*, 2011; Sato *et al.*, 2016; Wurm and Schubotz, 2018). To identify any brain region that might be related to social cognition, we in addition conducted *post hoc* whole-brain analyses. The initial voxel threshold was set to *P* < 0.001 uncorrected and multiple testing was accounted for on cluster level, based on a corrected pFWE < 0.05, as provided in SPM12. Stereotaxic coordinates are reported in MNI space.

## Results

### Demographic and clinical data

Participant characteristics are displayed in Table 1. Patients and healthy controls did not differ with regard to demographic variables such as body mass index or level of verbal intelligence (as assessed by MWT-B). However, there was a trend toward higher level of education in controls than in patients. As expected, patients scored significantly higher in questionnaires testing for current symptoms of ADHD (ADHS-SB), symptoms during childhood (WURS-k), as well as depressive symptoms (BDI). Scores of CAARS-S:L indicate overall high symptom severity in all symptom areas. However, performance in the executive functioning tasks did not differ between patients with ADHD and healthy controls. Note that four patients were medicated with stimulants when recruited for our study, but were asked to withdraw from their medication at least 48 hours before participation.

### Behavioral results

Descriptive data and statistical outcome values for all behavioral measures are depicted in Table 2. We did not observe significant differences in any of the outcome measures reflecting ToM performance (reduced ToM, affective ToM and cognitive ToM) between patients with ADHD and healthy controls (Mann–Whitney *U* test). There were also no group differences regarding the number of correct responses to the MASC control questions. The additional Bayesian analysis showed moderate evidence for the null hypothesis (absence of group differences) for all three outcome measures (BF_10_ < 0.3; Table 2). ToM performance did not correlate with ADHD scores (see Supplementary Material for correlation plots) or executive function performance (all *P* < 0.05; BF_10_ between 0.18 and 0.52).

*Post hoc*, we conducted additional analyses excluding patients receiving regular medication (*n* = 4). Further, we compared a subgroup of patients with high ADHD symptoms (median split based on ADHS-SB questionnaire: score > 32; *n* = 13) to their respective matched healthy controls. In both analyses, we did not find group differences in any outcome variable.

### VBM results

There were no significant differences in regional gray matter volumes between ADHD patients and healthy controls, in both ROI and whole-brain analyses.

**Fig. 1.**
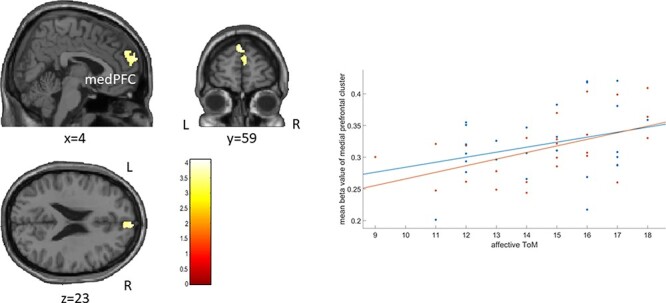
Correlation between affective ToM and gray matter volumes. Left: brain regions, in which gray matter volumes significantly correlated with affective ToM performance across groups of ADHD patients and healthy controls: medial prefrontal cortex (medPFC). Right: mean beta values of the medial prefrontal cluster correlating with scores for affective ToM in ADHD patients (red) and healthy controls (blue).

Across groups, higher affective ToM performance correlated with increased gray matter volume in one cluster comprising medial parts of the superior frontal gyri ([*x*, *y*, *z*] = [0, 59, 35]; *k* = 602; *r* = 0.43; 95% CI = [0.18; 0.63]; see Figure 1). In a *post hoc* whole-brain analysis, we observed an additional positive correlation between affective ToM and gray matter volume in one cluster in the right angular gyrus extending to right inferior parietal regions ([*x*, *y*, *z*] = [47, −60, 51]; *k* = 1035; *r* = 0.41; 95% CI = [0.15; 0.61]).

No other correlations between gray matter volumes and ToM outcome variables were observed.

There were also no group differences in total gray matter volume or total intracranial volume.

To control for the influence of possible confounding variables, the original analyses (group comparison and correlational analyses) were repeated, including depression scores (BDI) or gender as covariates. Additional models excluding patients receiving regular medication (*n* = 4) as well as including only patients with high ADHD symptoms (median split based on ADHS-SB questionnaire: score > 32; *n* = 13) and their respective matched healthy controls were also computed. Further, we conducted an analysis including only patients and their matched controls who were measured at the new scanner (Siemens MAGNETOM Prisma; *n* = 20 per group). Results of those analyses are presented in Supplementary Material.

## Discussion

In this study, we investigated social cognitive abilities and their brain structural correlates in adult patients with ADHD. Compared to a matched healthy control group, patients with ADHD did not show any impairments in ToM, as assessed by using the MASC. In addition, we did not observe any differences in gray matter volumes between ADHD patients and healthy controls. Adding relevant covariates to the analyses, excluding patients receiving regular stimulant medication or including only patients with higher ADHD symptom scores did not alter those findings. Across groups, affective but not cognitive ToM correlated positively with gray matter volumes in medial parts of the superior frontal gyri and the right angular and inferior parietal gyri. In other words, higher affective ToM performance was associated with increased gray matter volumes in these regions.

While previous studies have repeatedly demonstrated social cognition deficits in children with ADHD, results for adult patients are mixed. Our findings are consistent with the meta-analysis from Bora and Pantelis (2016), indicating no impairments in ToM in adults with ADHD. In addition, we replicate results of a recent study reporting no deficits in performance in the MASC in adult ADHD patients (Abdel-Hamid *et al.*, 2019). However, Abdel-Hamid *et al.* (2019) observed deficits in emotional aspects of empathy as assessed using the Cambridge Behaviour Scale in their patient sample, but unfortunately, they did not provide information on the affective and cognitive subscales of the MASC. In our study, ADHD symptoms were not associated with affective or cognitive aspects of ToM.

In addition, we observed no differences in brain morphological measures (neither in regional gray matter volumes nor in total gray matter or intracranial volume) between patients and healthy controls, which is well in line with the lack of ToM deficits in our ADHD sample. Our findings could be interpreted as support for the developmental delay theory in ADHD, suggesting that cortical development (in particular, in prefrontal regions) in adolescence is delayed in most patients (Shaw *et al.*, 2007), which might explain why social cognition deficits are commonly observed in children, but not in adults with ADHD. Previous investigations of brain structure and function in adult ADHD have revealed heterogeneous results, with some studies demonstrating neural abnormalities (Dickstein *et al.*, 2006; Schneider *et al.*, 2006; Makris *et al.*, 2007; Cubillo and Rubia, 2010; Wolfers *et al.*, 2017), while others suggest that brain abnormalities in ADHD patients decrease in adulthood (Shaw *et al.*, 2007; Nakao *et al.*, 2011; Frodl and Skokauskas, 2012; Hoogman *et al.*, 2017, 2019). Our findings are in line with a mega-analysis by the ENIGMA ADHD Working Group (Hoogman *et al.*, 2017), which included 23 cohorts with 1713 patients with ADHD and 1529 healthy controls. They showed that the smaller subcortical and total intracranial volumes found in ADHD patients were mainly due to the effects in children, while no differences between adult patients and controls were observed. Notably, our study is the first relating brain structural correlates to social cognition abilities in adults with ADHD. One previous study in children with ADHD identified a relation between smaller subcortical volumes and deficits in ToM and social skills, but this association also decreased with increasing age (Baribeau *et al.*, 2019).

In our adult sample, superior skills in emotional aspects of ToM (i.e. inferring affective states) were associated with larger volumes in medial parts of the superior frontal gyri, and in whole-brain analyses, with larger volumes in the right angular gyrus and inferior parietal regions. This is in line with previous studies showing associations between gray matter volume in those regions and social cognition in healthy and clinical populations (Abu-Akel and Shamay-Tsoory, 2011; Sato *et al.*, 2016; Pera-Guardiola *et al.*, 2016). In addition, reduced gray matter volumes, in particular, in the medial prefrontal cortex have often been reported in children and adults with ADHD (e.g. Bralten *et al.*, 2016), and it is conceivable that reductions in frontal areas might contribute to social deficits. However, our patient samples showed neither impairments in ToM nor structural alterations compared to the healthy control group. Moreover, we did not identify any structural correlates for cognitive perspective taking (i.e. understanding of thoughts and intentions), which is not fully explicable. Previous work has suggested that affective ToM and cognitive ToM rely on distinct, but also overlapping brain regions. In meta-analyses and systematic reviews of functional MRI studies, affective ToM is frequently reported to be associated with stronger activation in medial prefrontal regions, although those regions are also relevant for cognitive ToM (Jankowiak-Siuda *et al.*, 2011; Bzdok *et al.*, 2012; Molenberghs *et al.*, 2016; Stietz *et al.*, 2019). Studies on the structural correlates of cognitive and affective aspects of ToM are scarcer and provide rather heterogeneous results. Using the MASC, Laillier *et al.* (2019) investigated the structural correlates of ToM in healthy older adults and observed correlations between scores for affective ToM and volumes in several regions of the frontal, parietal and temporal cortices, including superior medial frontal areas. In their study, cognitive ToM also correlated with volumes in frontal regions, which were, however, localized more laterally. Note that in older adults, decline is rather observed in cognitive components of ToM, while those studies implicating social cognition deficits in adults with ADHD observe those rather in emotional domains (e.g. Bora and Pantelis, 2016; Abdel-Hamid *et al.*, 2019).

Notably, in our study, we excluded severe comorbidities (e.g. autism spectrum disorders, substance abuse, severe anxiety disorders and severe depression) and one-half of our patient sample was free of any psychiatric comorbidities. However, half of the patients showed comorbid mild-to-moderate depression and/or anxiety disorder, and one patient presented comorbid borderline personality disorder. While this heterogeneity in our sample has to be considered when interpreting our findings, including patients with frequently occurring comorbidities has the advantage of a more representative ADHD sample (e.g. Heidbreder, 2015). Importantly, the present comorbidities have all been associated with deficits in social cognition (e.g. Plana *et al.*, 2014; Patin and Hurlemann, 2015; Bora and Berk, 2016; Domes *et al.*, 2019) and alterations in brain structure (e.g. Koolschijn *et al.*, 2009; Krause-Utz *et al.*, 2014; Kolesar *et al.*, 2019) in previous studies. In this perspective, the lack of differences in social cognition or brain structure between patients and controls in our study appears all the more interesting.

Although ADHD symptom severity in our study was high on average (see Table 1, CAARS-S:L) and impairments in self-concept as an indicator of social difficulties were present in most patients, approximately one-third of the patients showed rather low-to-moderate symptoms. However, subgroup analyses of patients with higher symptom scores did not yield ToM deficits as well. In addition, on group level, patients did not show any impairments in executive functioning, and previous studies have indicated a close association between executive functions and social cognitive skills. Mary *et al.* (2016) even suggested that poor executive functioning may account for ToM deficits in ADHD. The overall high heterogeneity in previous findings regarding social cognition as well as brain abnormalities in adults with ADHD might be partly caused by different characteristics of the included samples such as comorbid disorders and/or areas of impairment (see, e.g., Bora and Pantelis, 2016). Ibanez *et al.* (2014) for instance found no ToM deficits in a small group of adult ADHD patients, who did not present with any comorbidities or executive function deficits. On the other hand, Abdel-Hamid *et al.* (2019) also excluded patients with psychiatric comorbidities and still observed impairments in executive functioning and emotional empathy, but not in ToM.

Finally, it is worth noticing that we cannot exclude the possibility that the lack of differences between patients and healthy controls in our study might be due to the small sample size. Because data acquisition was conducted in the context of a larger project on the effects of exercise on cognitive functions in ADHD (including behavioral outcomes and fMRI), power analyses were conducted for the primary outcome parameters of this project. To detect differences in social cognitive skills between patients with ADHD and healthy controls, for which small-to-moderate effect sizes would be expected (e.g. Bora and Pantelis, 2016; Abdel-Hamid *et al.*, 2019), a greater sample would be clearly favorable. On the other hand, our sample size is in the range of previous studies on social cognition and/or brain abnormalities in ADHD. In light of the still very scarce literature in adult patients with ADHD, we believe that this study can nevertheless contribute to the advancement of this field and can inform future research. Consequently, in future studies, it would be interesting to recruit larger patient samples, including patients with a wide range of symptom severity and areas of impairment, to investigate the neuroanatomical correlates of (potential) social cognition deficits in those patients. Moreover, a combination of multiple measures assessing distinct aspects of social cognition (e.g. self-assessments/questionnaires and neuropsychological tests) might provide further insights into possible social cognitive shortcomings in adults with ADHD.

## Conclusions

Social cognition deficits have been frequently reported in children with ADHD, while findings for adult patients are mixed. In our adult ADHD sample, we did not observe any impairments with regard to affective or cognitive aspects of ToM. In addition, we did not find structural differences in brain regions typically underlying social cognitive abilities between patients and healthy controls, which might provide a potential neural basis for the lack of social cognition deficits in our sample. However, to interpret such findings in light of the view that ADHD is characterized by a delay in cortical development, longitudinal studies to relate social cognitive development to brain maturation are necessary.

## Supplementary Material

nsab060_Supp

## Data Availability

The datasets generated and analyzed during the current study are available from the corresponding author on reasonable request.
