# Supplementary material for: Unimpaired social cognition in adult patients with ADHD: brain volumetric and behavioral results
Source: Soc Cogn Affect Neurosci. 2021 May 7;16(11):1160–9. doi: 10.1093/scan/nsab060 (PMC8599175; doi:10.1093/scan/nsab060)
Supplement: nsab060_Supp [file nsab060_supp.zip › Supplementary_Material.docx]

***Supplementary Material***

**Unimpaired social cognition in adult patients with ADHD: brain volumetric and behavioral results**

Mehren A, Thiel CM, Bruns S, Philipsen A, Özyurt J

*Mean values for behavioral outcomes and demographic/clinical data*

**Table S1.** Mean values with standard deviation (SD) for demographic/clinical characteristics and performance in the Movie for the Assessment of Social Cognition (MASC) among patients with ADHD and healthy controls

| **Variable** | **ADHD**  **mean (SD)** | **Controls**  **mean (SD)** |
| --- | --- | --- |
| Age (years) | 31.1 (9.3) | 30.2 (8.5) |
| BMI (kg/m²) | 25.8 (4.1) | 24.2 (4.0) |
| Years of education | 13.6 (2.6) | 15.0 (2.6) |
| MWT-B | 27.7 (3.3) | 29.3 (3.6) |
| ADHS-SB | 31.7 (8.5) | 4.7 (4.0) |
| WURS-k | 41.9 (14.5) | 10.0 (7.3) |
| CAARS-S:L (T-scores) |  |  |
| Inattention/Memory problems | 72.3 (13.5) | --- |
| Hyperactivity/Restlessness | 69.9 (11.7) | --- |
| Impulsivity/Emotional lability | 68.1 (10.8) | --- |
| Problems with self-concept | 65.5 (15.7) | --- |
| DSM-IV inattentive symptoms | 72.5 (13.8) | --- |
| DSM-IV hyperactive/impulsive | 69.3 (10.9) | --- |
| DSM-IV total symptoms | 74.3 (12.4) | --- |
| ADHD-index | 74.7 (11.1) | --- |
| BDI | 10.0 (7.1) | 2.0 (2.3) |
| Go/No-go sensitivity | 3.2 (0.6) | 3.4 (0.6) |
| Flanker interference (ms) | 76 (22) | 81 (19) |
| Reduced ToM | 6.2 (3.7) | 6.2 (3.0) |
| Affective ToM | 14.4 (2.6) | 14.6 (2.1) |
| Cognitive ToM | 17.6 (2.7) | 17.5 (2.6) |
| Exceeding ToM | 4.4 (2.2) | 4.6 (2.5) |
| Correct ToM | 34.3 (4.7) | 34.3 (4.0) |
| Correct control questions | 4.6 (0.9) | 4.8 (1.0) |

ADHD: N = 26 (5 females); Controls: N = 26 (5 females). BMI = body mass index; MWT-B = Multiple Choice Vocabulary Test; ADHS-SB = ADHD Self Rating Scale; WURS-k = Wender Utah Rating Scale, retrospective assessment of childhood ADHD; CAARS-S:L = Conners’ Adult ADHD Rating Scale–Self-Report: Long Form; BDI = Beck Depression Inventory; ToM = theory of mind.

*Correlational analyses between theory of mind (ToM) and ADHD scores*

**
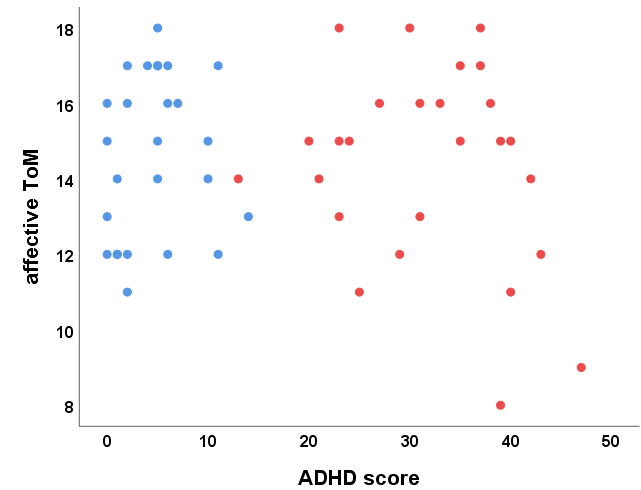

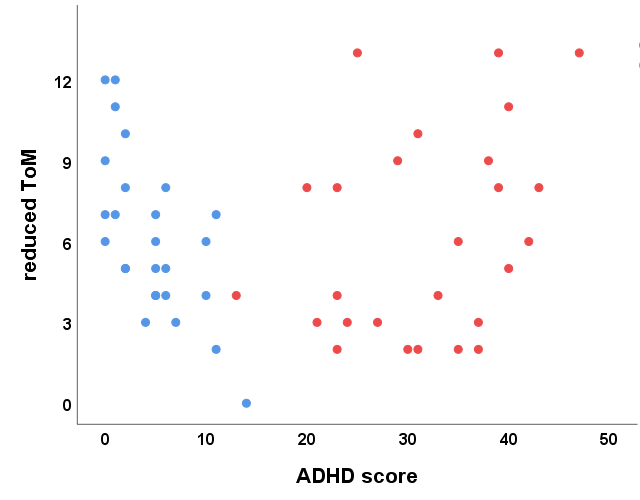
**

**
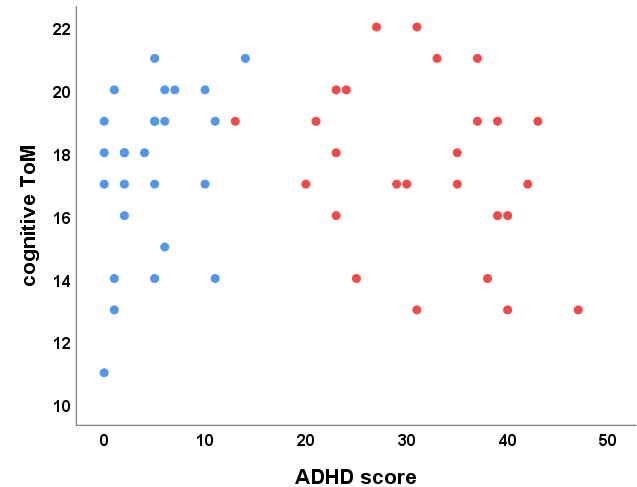
**

**
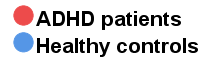
**

**Figure S1.** Correlations between theory of mind (ToM) performance and ADHD Self Rating Scale (ADHS-SB) across both groups (all p > .05).

*Additional voxel-based morphometry (VBM) analyses*

To control for the influence of potential confounders on study outcomes, we computed the original VBM analyses (group comparison and correlational analyses) again including depression scores (BDI) or gender as covariates.

In all models using both, ROI and whole brain analyses, we did not observe any significant differences in regional gray matter volumes between ADHD patients and healthy controls.

The positive correlation observed in ROI analyses across both groups between affective ToM performance and gray matter volumes in the medial frontal cortex was significant on trend level when adding depression scores as covariate ( [x, y, z] = [0, 58, 34]; k = 526; p = .057). In post hoc whole brain analyses, the additional positive correlation between affective ToM and gray matter volume in the cluster extending from the right angular gyrus to right inferior parietal regions remained significant ([x, y, z] = [46, -60, 51]; k=948; p=.037). Including gender as covariate to the original model resulted in no significant correlations. As in the original models, no other correlations between gray matter volumes and ToM outcome variables were observed. Gray matter volumes did also not correlate with self-reported ADHD scores (ADHD Self Rating Scale, ADHS-SB) or age.

Excluding patients receiving regular stimulant medication and their respective matched healthy controls (n = 4 per group) as well as comparing patients with higher ADHD scores (ADHS-SB score > 32; n = 13) to their matched controls did not reveal any significant differences in regional gray matter volumes in the predefined ROI. Including only participants who were measured at the Siemens Magnetom Prisma scanner (n=20 per group) did also not reveal any group differences.
